# Use of Transfer Learning for the Automated Segmentation and Detection of Swallows via Digital Cervical Auscultation in Children

**DOI:** 10.1007/s00455-025-10833-3

**Published:** 2025-06-03

**Authors:** Stephen So, Timothy Tadj, Belinda Schwerin, Anne B. Chang, Thuy T. Frakking

**Affiliations:** 1 Research Development Unit, Caboolture Hospital, Metro North Hospital & Health Service, McKean St, Caboolture, QLD 4510 Australia; 2 Child Health Research Centre, School of Medicine, The University of Queensland, South Brisbane, QLD 4101 Australia; 3 Speech Pathology Department, Gold Coast University Hospital, Gold Coast Hospital & Health Service, 1 Hospital Boulevard, Southport, QLD 4215 Australia; 4 School of Health Sciences & Social Work, Griffith University, 1 Parklands Drive, Southport, Gold Coast, Queensland 4222 Australia; 5 School of Engineering and Built Environment, Griffith University, Parklands Dr, Southport, QLD 4215 Australia; 6 Department of Respiratory Medicine, Queensland Children’s Hospital, 501 Stanley St, South Brisbane, QLD 4101 Australia; 7 Child Health Division, Menzies School of Health Research, Charles Darwin University, PO Box 41096, Casuarina, Northern Territory 0811 Australia; 8 Australian Centre for Health Services Innovation, Queensland University of Technology, Level 7, 62 Graham St, South Brisbane, QLD 4101 Australia

**Keywords:** Cervical auscultation, Deglutition, Swallowing sounds, Signal processing, Machine learning, Pediatric

## Abstract

Digital cervical auscultation (CA) has high diagnostic test accuracy in the detection of aspiration in children. However, the clinical application of digital CA is limited because swallow sound recordings require manual segmentation by trained experts, which is time consuming and not feasible in clinical practice. The automated detection of swallowing sounds in adults from sound recordings have reported accuracies between 76 and 95%. No equivalent literature exists for the automated detection of swallowing sounds in children. This study aimed to establish whether automated machine learning using a transfer learning approach can accurately detect and segment swallows from digital CA recordings in children. Swallow sounds were collected from 16 typically developing children, median age 18 months (range 4–35 months, 50% males); and 19 videofluoroscopic swallow studies of children with pediatric feeding disorders, median age 9 months (range 3–71 months, males 36.8% males). All swallowing sounds were on thin fluids. A deep convolutional neural network (DCNN) that was pre-trained for the task of audio event classification was used as the base machine learning model. Using the raw swallow audio data as input, embedding vectors from the base DCNN were computed and used to train a feedforward neural network to identify whether an audio segment was a swallow or not. A high overall accuracy of 91% was achieved using our model, with a sensitivity (or recall) and positive predictability (or precision) of 81% and 79%, respectively. Interestingly, the model was also able to detect saliva swallows in the clinical feeding evaluation test set, even though these non-nutritive swallows were not part of the training set. This indicates a level of generalizability of the model, where it was able to recognize swallowing events that it had not “seen” before. Our study provides the highest accuracy reported to date on the automatic segmentation and detection of swallowing sounds in children.

**Supplementary Information:**

The online version contains supplementary material available at 10.1007/s00455-025-10833-3.

## Introduction

Aspiration, the entry of food/fluids or foreign bodies into the airway, is common among pediatric feeding disorders, with prevalence ranging between 34% [1, 2] in hospitals and up to 90% in children with neurological and developmental disorders [3]. The early detection of aspiration in children is important to prevent consequences of acute and chronic lung diseases [4], poor growth and development and high caregiver stress [5]. A videofluoroscopic swallow study (VFSS, a dynamic x-ray swallow study) is the current gold standard for assessing aspiration in children [6]. However, the repeatability of VFSS is limited because it can only be utilized 1–2 times per year due the risks of cumulative exposures to ionizing radiation; [7, 8] and the procedure may not replicate typical mealtimes (e.g. barium added with food/fluids;[9, 10] unfamiliar environment of medical imaging suite) [11]. A complementary assessment to the VFSS that is repeatable, cost-effective and equivalent in aspiration detection accuracy to the VFSS has the potential to enhance access to assessments across healthcare facilities worldwide, particularly in regional, rural and less wealthy regions without direct access to VFSS.

Cervical auscultation (CA), a technique that detects cervical sounds generated during the swallow and breath sounds pre- and post-swallow, is routinely used by approximately one-third of speech pathologists in the United Kingdom, Ireland and Australia [12, 14]. Its continual use and appeal to speech pathologists in clinical practice are likely related to its availability, repeatability and increased ability to accurately detect clinical signs of aspiration. Compared to VFSS, CA has high sensitivity (0.85–0.93), [15, 17] very good inter-(kappa 0.81) and intra-rater reliability (kappa 0.72–0.98) [17] in detecting aspiration in children. Recently, we found that machine learning classifiers from specifically segmented audio from recorded swallowing sounds demonstrated 89% and 100% sensitivity in the classification of aspirating (*n* = 9) and normal swallows (*n* = 53) in children [11]. As the segments above used clean annotated and labelled segmented audio, the technique cannot, as yet, be automated for it to be applied into clinical practice for two main reasons: (a) the sound recordings differ significantly in terms of duration and amount of extraneous noises typically heard in clinical practice; and (b) swallow sound data relies on time-consuming expert manual segmentation of the recording which is not feasible outside of research settings. A machine learning algorithm that automatically segments and detects swallows based on real-world CA data potentially eliminates these barriers and provides an important first step for the use of transfer machine learning to detect swallows.

The automatic detection of swallowing sounds has been limited by a lack of standardization to enable comparative evaluation and validation [18]. Works investigating the segmentation of swallowing sounds recorded via microphone typically apply strategies founded in automatic speech recognition and speech processing. Approaches include thresholding of selected acoustic or spectral features, [19, 21] Mel Frequency Cepstrum Coefficients (MFCC) as input to a Hidden Markov Model (HMM), [22] variations on MFCCs in a Gaussian Mixture Model (GMM), [23] Mel-scale Fourier spectral features in a support vector machine (SVM), and frequency domain dynamic time-warping template matching [24]. These works aim to segment swallows in adult patients, compare to manually identified swallow segments, and had mixed experimental conditions (e.g. saliva, fluid and food swallows). More recently, Kimura and colleagues [25] recently reported an accuracy of 95.2% in the automated detection of swallow sounds when a variety of acoustic features were applied. However, this study, rather than automatic segmentation, looked at classifying loud acoustic events as “swallows” or “non-swallows”, where the loud acoustic event segments were determined using an arbitrary threshold of the logarithmic energy of each frame. Neural Network and Deep Learning based approaches have also been proposed. Aboofazeli and colleagues [26] used spectral features as input to a 2 layer feedforward Neural Network (NN) on a small data set of tracheal sound recordings from healthy adult participants, reporting an average accuracy of 91.7%. However, due the limited number of subjects, a jackknife sampling was used and it was not possible to ensure that the participants being tested, did not have their other swallows appearing in the training. More prevalent are works focused on the segmentation of accelerometry data, noting improved accuracy over that from sound recordings. Khalifa and colleagues [27] used segment spectrograms as input to a “Deep Neural Network” (DNN) with two hidden layers of 1285 units each, noting 89% average accuracy on clean swallows and 76% across whole dataset of A-P accelerometer recordings and adult participants. Anecdotally in clinical practice, however, accelerometry recordings and subsequent manual segmentation of swallow sounds are not routinely completed by clinicians due to the increased time required to complete this process. Furthermore, the study used the training set as validation to determine the optimal window size, which can often lead to overfitting. In addition, the main study did not appear to ensure that a patient’s data was not cross contaminating both training and test sets, which can reduce the generalizability of the model to unseen patient swallows. Further, works surveyed considered adults, either healthy or with specific conditions, rather than paediatric participants, and swallowing labels of data used in training and testing were manually determined, rather than by using gold standard VFSS.

In this paper, we present a machine learning-based system that automatically segments a digital audio recording into swallowing and non-swallowing sounds. Our approach uses transfer learning, where a pre-trained deep learning model for classifying hundreds of audio sounds is adapted to the task of identifying swallow sounds. The difficulty with training deep neural network models is that enormous amounts of human-labelled data are often required to avoid low generalization performance and overfitting. Transfer learning is a powerful tool since it allows a deep learning model, which has been trained on large amounts of data, to enhance the performance in a similar classification task that may have only a limited amount of labelled data. This means that information that was learnt in the previous task can be reused to help boost the performance in the new task. This contrasts with previous studies, which due to limited amount of data, have employed shallower learning methods, such as multilayer perceptrons [25], two hidden layer neural networks [26, 27] and support vector machines [25]. In our proposed model, we utilize the publicly available YAMNet model [28] for audio classification. YAMNet is a deep convolutional neural network that has been pre-trained to predict 521 different types of audio events from the AudioSet dataset [29], which consists of 2,084,320 human-labelled 10 s audio clips of variable quality from YouTube videos. These sounds cover everyday environment sounds, human and animal sounds, and musical instruments. While the YAMNet model was not specifically designed for the purpose of detecting swallow sounds, it has been successfully used in other studies (e.g. lung sounds, audio interval retrieval) that have achieved accuracies of 89–92% [30, 31]. The idea is that acoustic features and embeddings that were learnt in YAMNet for discriminating a wide range of audio classes would be useful for identifying, amid other environmental sounds, the distinct audio events associated with swallowing. Thus, the primary aim of this study was to establish whether automated machine learning using a transfer learning approach can accurately detect and segment swallows from digital CA recordings in children.

## Methods

A Human Research Ethics Committee (HREC) approved the additional data analyses on swallowing sounds previously collected from children in two separate studies (HREC/11/QRCH51 and HREC/11/QRCH52).

### Participants

Thin fluid bolus swallowing sounds collected from feeding observations of 16 typically developing children, median age 18 months (range 4–35 months, 50% males); and 19 VFSS’ of children with pediatric feeding disorders, median age 9 months (range 3–71 months, males 36.8% males). In depth details of the inclusion and exclusion criteria for typical developing children [32] and children with paediatric feeding disorders [15, 16] are available in published studies. Children with feeding disorders had a range of underlying etiologies including Beckwith-Wiedermann syndrome (*n* = 1), Cru de Chat (*n* = 1), Pierre Robin Sequence (*n* = 1), spinal muscular atrophy (*n* = 2), cerebral palsy (*n* = 2), bronchiectasis (*n* = 1), chronic cough (*n* = 1), oesophageal atresia (*n* = 2), congenital myopathy (*n* = 1), anemia (*n* = 1), tracheoesophageal fistula/airway anomalies (*n* = 2), developmental delays (*n* = 1) or failure to thrive (*n* = 1). Two children had no known underlying medical diagnoses.

### Procedure

Thin fluid bolus swallow sounds from typically developing children.

This dataset consisted of sixteen children drinking thin fluids extracted in one minute visual-audio recordings from mealtime observations, totalling 283 swallows. Children were seated upright at a small chair and table; in a high chair; or semi-reclined on their caregiver’s lap. Open or spout cup drinking was observed in 11 children and 5 children were breast or bottle-fed thin fluids.

Thin fluid bolus swallow sounds from VFSS in children with pediatric feeding disorders.

This dataset consisted of one minute visual-audio recordings extracted from 19 VFSS’ of children with pediatric feeding disorder drinking thin fluids, totalling 275 swallows. Children were seated semi-reclined on a tumbleform chair or independently sat upright on a Videofluroscopic Imaging Chair. The lateral view was used for all VFSS recordings. A range of drinking methods were used including bottle/teat (*n* = 8), open or spout cup (*n* = 7), straw (*n* = 2) and syringe feeding (*n* = 2).

### Equipment

An omnidirectional condenser microphone (C417, AKG Acoustics, Vienna, Austria) (sensitivity at 1 kHz of 10 mV/Pa, impedance 200, frequency range 20 to 20,000 Hz) was used to record all swallow sounds. The microphone, which was inserted into a fitted O-ring was positioned on the neck by palpating the cricoid cartilage and secured laterally (< 1 cm) to this position using microfoam tape by principal researcher (TTF) to ensure consistency of placements. The microphone was connected to a Digital H4n Handy Recorder (Zoom Corporation, Tokyo, Japan). Sound recordings were checked for quality prior to and throughout the recordings using headphones (Model ATH-M50, Audio-Technica, Taiwan). Where a reduction in sound quality was noted (e.g. loss of microphone contact to skin surface), adjustments to the microphone placement were made.

A digital video-recorder (Model DCR-DVD605E, Sony Corporation, Tokyo, Japan) was used to capture mealtime observations of typically developing children drinking thin fluids. VFSS procedures for children with pediatric feeding disorder were captured at 15 frames per second using a digital fluoroscopy unit (Toshiba KXO-80G, North Ryde, NSW, Australia). All visual recordings were recorded on the Digital Swallowing Workstation (KayPentax, Pentax, New Jersey, USA). To facilitate later synchronization of acoustic and visual data, a vocal signal “go” was expressed by TTF at the beginning of all visual-audio recordings. Sony Vegas Movie Studio 9 (Madison, WI) was used to synchronize all visual and audio recordings. The timepoints for swallow sounds were manually segmented by TTF, a pediatric speech pathologist with postgraduate doctoral skills in cervical auscultation. The beginning of bolus swallow sounds were characterized by the start of a fluid flushing sound (audio data) and initial laryngeal movement (visual data). The end of bolus swallow sounds were characterized by the absence of a fluid flushing sound (audio data) and no laryngeal movement (visual data). Saliva swallow timepoints from mealtime observations were marked when a bolus transit sound was audible in the absence of any ingestion of thin fluids (e.g. saliva swallows before the first sip of thin fluids). It was outside the scope of this study to segment non-swallows, which meant that only two classes (e.g. swallows and non-swallows) were used for classification. Non-swallows consisted of sounds that were not considered as swallows (e.g. background noise, coughing, talking).

### Data Integrity, Preprocessing and Feature Extraction Stage

**Fig. 1:**
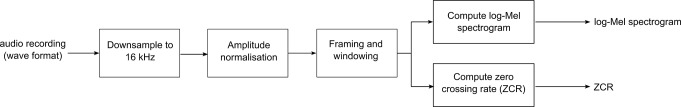
Block diagram of feature extraction stage

Electrical and electronic engineers (BC and SS) listened to an initial sample of *n* = 10 audio files and visually inspected waveforms for the assessment of data integrity. Both engineers determined that the initial samples assessed were within normal limits of amplitude (e.g. no clipping of sound, swallow sound volume was audible above background noise). Additionally, the preprocessing and feature extraction of the audio data was completed by both SS and BS, which is summarised in Fig. 1. In order to match the sampling rate used in the YAMNet model, the digital audio files were downsampled from 44.1 kHz to 16 kHz using appropriate signal processing techniques. The audio data was then normalized so that the amplitude values occurred between − 1 and 1.

The audio data was then split into frames of 0.96 s with a 50% overlap. Each frame of audio data was converted into a log-Mel spectrogram and these were used as the input audio features to the pre-trained YAMNet model. The mel-scale warping of the frequency is a speech signal processing method that is used to simulate the non-linear frequency resolution of the inner human ear, while the logarithmic operation on the Mel-scale filter outputs simulates the amplitude compression applied by the middle ear [33].

### Embedding-Feature Vector Concatenation

While the base deep convolutional model used log-Mel spectrograms as the input feature, we have found through our experiments on swallowing sound detection that further performance gains were able to be achieve by incorporating extra audio features used in automatic speech recognition. Of the several feature vectors typically used, the zero-crossing rate (or ZCR) was found to provide noticeable gains in classifier performance while being computationally simple to compute. The ZCR measures the rate at which the waveform changes its sign (i.e. crosses the zero axis) and has been used as a feature in automatic speech recognition for estimating the dominant frequency in the signal [31].

Complementing the log-Mel spectrogram features, the ZCR feature was computed for each frame and concatenated with the embedding vector, rather than the log-Mel spectrogram input. Further details are provided in the next section.

### Transfer Learning and the Final Neural Network Model

**Fig. 2:**
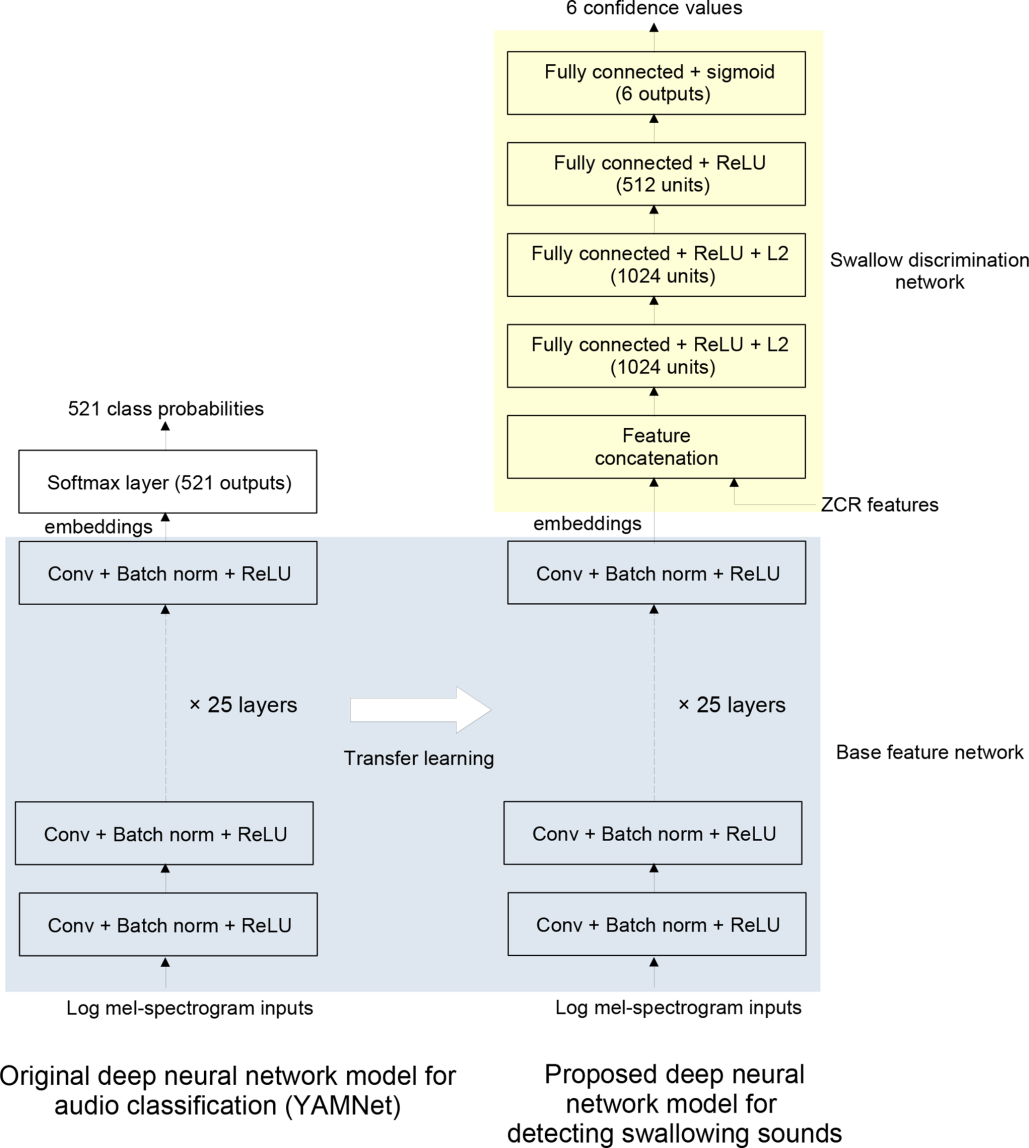
Block diagram of the transfer learning and final inference model

The original YAMNet model consists of multiple convolution layers for feature extraction, comprising 28 layers of full, depth-wise separable, and point-wise convolutional layers. Each layer was followed by batch normalisation to handle covariate shift [34] and the rectified linear unit (or ReLU) [35] activation function. The final layer consists of a softmax classification layer. In the transfer learning approach used in this study, the YAMNet embeddings (i.e. the outputs from the top-most convolution layer that feed into the softmax layer) were computed for each data frame. As shown in Fig. 2, these embeddings were then concatenated with the ZCR feature computed for that frame. The softmax layer of YAMNet was then replaced with a fully-connected neural network (FCNN) consisting of three hidden layers (with 1024, 1024 and 512 units, respectively) and an output layer of six units. The ReLU [35] activation function was used in the input and hidden layers of the FCNN, while the sigmoid activation function was used in the output layer. To mitigate model overfitting, L2 regularisation was applied to the first two hidden layers.

### Improving the Temporal Resolution of the Segmentation Using Sub-Frame Prediction

**Fig. 3:**
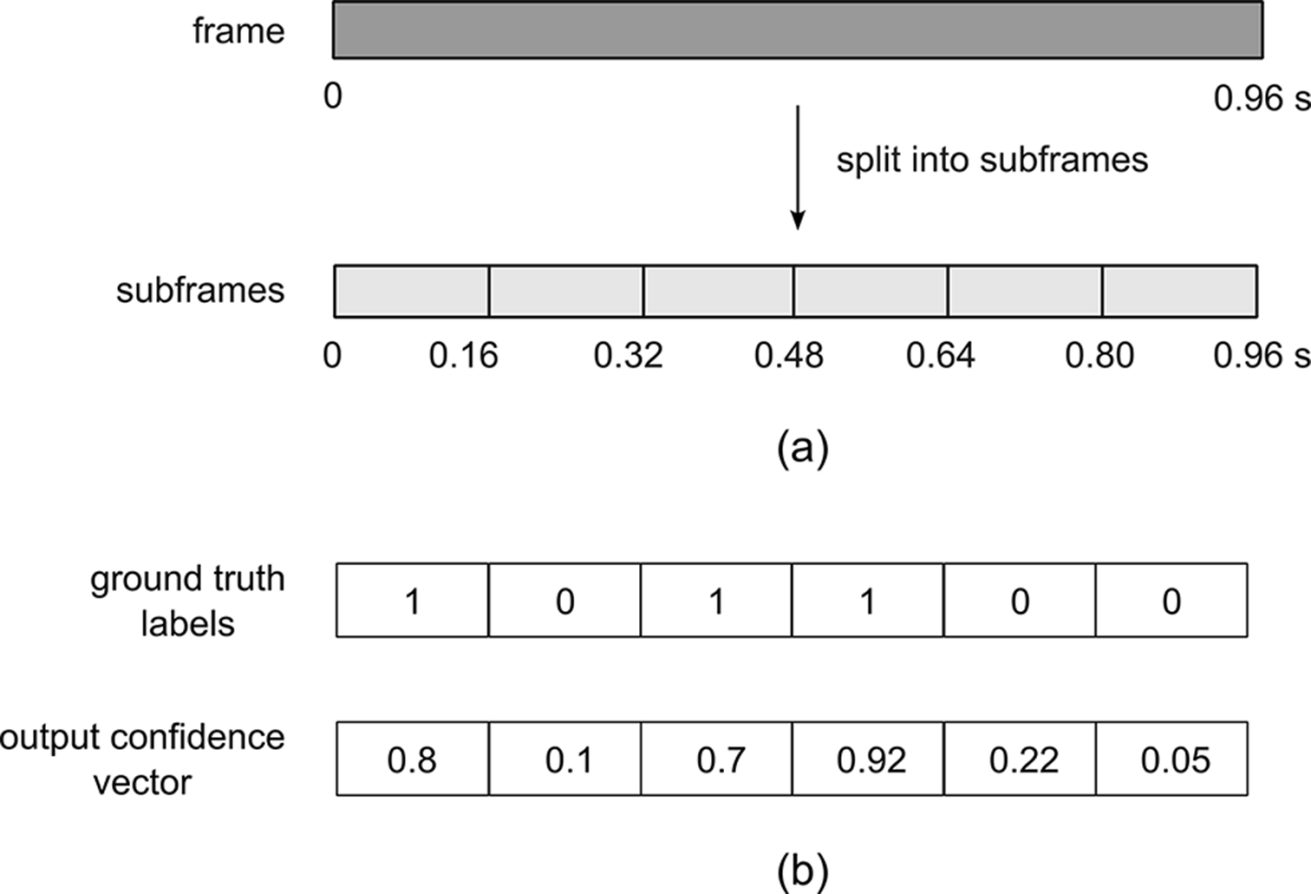
Increasing temporal resolution of segmentation. (**a**) Each 0.96 s frame is split into six subframes of 0.16 s duration; (**b**) example of a ground truth label and model output confidence vector showing swallows occurring in subframes 1, 3 and 4

As shown in Fig. 3 (a), in order to increase the temporal resolution of the segmentation, each long frame was further subdivided into six smaller sub-frames, each being 0.16 s in duration. In the ground truth label (Fig. 3 (b)), the occurrence of a swallow in each subframe was represented by a 1, while non-swallows were assigned the value of 0. The six-unit output layer of the FCNN produced a vector of six confidence values of between 0 and 1. The predicted confidence values for each long frame are overlapped, added, and the final predictions were obtained via a threshold. Through this subframe prediction mechanism, the temporal resolution was improved by a factor of six, i.e. it was able to detect swallowing events that occurred within a window of 0.16 s.

### Experimental Setup

The recordings were presented to the model as 16-bit wave files, one for each participant. The ground truth labels, in the form of start and stop times for each swallow, were recorded in a Microsoft Excel spreadsheet. Python and its associated libraries (numpy, scipy, pandas, scikit-learn, tensorflow, etc.) was used as the software platform to implement the model training and evaluation experiments.

Participants were separated to ensure no patient overlap across the training, validation and testing datasets. The participants in the VFSS dataset were divided into training and validation subsets in an 80/20 ratio, where great care was taken to ensure that participants used for the training subset, were not used in the validation subset. The test dataset consisted of recordings from mealtime observations only. The data was randomly balanced and shuffled prior to each training epoch to ensure an equal proportion of swallowing and non-swallowing sounds were available to the model. The training process adopted in this study used mini-batches of size 32 and utilized the Adam optimizer with the mean squared error (or MSE) as the loss function. Only the weights in the FCNN were adjusted during the training. A maximum of 100 epochs was set for the training. The Google Tensorflow 2.15 software was used to implement the machine learning model and training and inference were run on a PC with an AMD Ryzen Threadripper 2950X CPU and equipped with an NVIDIA RTX A6000 graphics card.

## Results

**Fig. 4:**
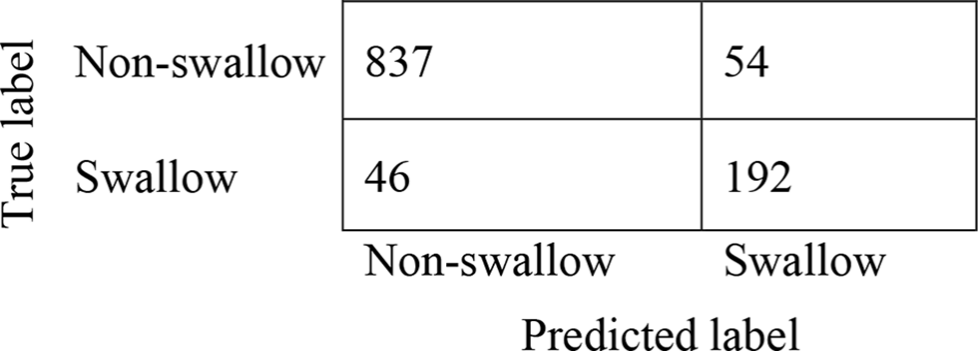
Confusion matrix between non-swallow and swallow classes in mealtime observations dataset

The confusion matrix of the non-swallow (0) and swallow (1) classes are shown in Fig. 4. A true positive for a swallow was recorded if at least 30% of the swallowing event was correctly predicted by the model. The fraction of 30% was chosen based on Kimura et al., [25] where an audio event was classified as a swallow if it overlapped with at least 35% of the true swallow. The confusion matrix shows that of the true non-swallows, the classifier correctly identified 837 frames as non-swallows, and 192 as swallows. Of the true swallows (*n* = 232), where five were identified and validated as saliva swallows, the classifier labelled 46 as a non-swallows and 192 as swallows (sensitivity, 81%). Of the true non-swallows (*n* = 891), the classifier labelled 847 as non-swallows and 54 as swallows (sensitivity, 94%). Table 1 lists the overall performance of the model, where the total accuracy was found to be 91%. Supplemental Figs. 1 and 2 show the waveforms of the locations for (a) true labelled swallows; and (b) model-predicted swallows in participants used in the validation and testing datasets. Supplemental Fig. 3 shows a zoomed-in five-second window of a participant used in the testing datasets, showing finer detail of the swallowing events that were detected.

**Table 1:** Model performance between patients with non-swallow and swallows in the test set (total accuracy = 0.91)

	PPV or Precision	NPV	Specificity	Sensitivity or Recall	F_1_-score
Non-swallow (0)	0.95	0.79	0.81	0.94	0.94
Swallow (1)	0.79	0.95	0.94	0.81	0.79

Abbreviations: PPV = positive predictive value; NPV = negative predictive value. The F_1_ score, which is defined as the harmonic mean of the precision and recall, is another measure of accuracy that is more conservative, balances the contribution of false negatives and false positives to the final metric, and is better suited to cases where classes are unbalanced

## Discussion

To our knowledge, this is the first study to use transfer learning to build a neural network that segments swallowing sounds in an audio recording. The premise of transfer learning is to leverage an existing neural network model that has been pre-trained on a much larger amount of data for a similar category of machine learning task. In our case, we have used the YAMNet model, [28] which was trained to classify human-labelled audio from YouTube videos. The hypothesis is that YAMNet has learnt higher level representations from the input speech recognition features, i.e. Mel-scale-warped spectrograms, in its convolutional network layers that are useful for discriminating between audio classes. Therefore, these “learned” higher level features were transferred to the swallow segmentation task in the form of inputs to a custom feedforward neural network that has been trained for the task of discriminating swallowing sounds. This approach allows us to overcome the problem of the lack of training data that is necessary to train deep neural networks.

The high accuracy achieved using our model (91%), with sensitivities of 81% and 94%, for swallowing and non-swallowing sounds, respectively, validates our approach. The robustness of the model to mismatched data, where we have trained on participants on a VFSS-labelled dataset and tested it on clinical feeding evaluations dataset, is apparent in the results. This contrasts with the study by Khalifa et al., [27] where an average accuracy of 89% was reported on A-P accelerometer data with overlapping patients amongst the training and test sets. We infer also that the audio recordings we used were not as clean as the accelerometer recordings used in the study by Khalifa et al., [27] since accelerometer sensors generally are less sensitive to and have fewer types of undesirable noise when compared with the condenser microphones used in our study, which are known to be highly sensitive to all types of environmental noise in the clinical setting. Furthermore, there was no further postprocessing or “temporal enhancement” process of the output mask from our model. Our model was trained on gold standard VFSS recorded swallow sounds and tested on clinical feeding evaluations, which is reflective of swallow sounds heard in clinical practice. Given that the clinical feeding evaluations dataset was completely unseen until the testing phase, we hypothesize that the high accuracy found is generalizable to clinical practice. Additionally, the model was able to detect saliva swallows which provides promising evidence that the model is able to generalize to detect saliva swallows in addition to bolus swallows. Also, there was sufficient mismatch between the training and testing datasets to account for differing recording conditions for each individual patient.

Our overall sensitivity of 81% included the presence of false negatives that is likely due to the machine learning detection of spontaneous saliva swallows in the testing set. This type of swallow was not manually segmented and classified in the VFSS training and validation datasets because our study focussed on volitional bolus swallows of thin fluids in children only. Previous swallow detection and segmentation classifiers in adults and children have categorized swallowing and non-swallowing sounds (e.g. spontaneous saliva swallows, cough, voice) [36]. We question the relevance of classifying non-swallowing sounds such as voice because of its vastly different acoustic characteristics compared to swallow sounds; and the clinical relevance of accurately classifying these sounds during a clinical feeding examination. Spontaneous saliva swallows, on the other hand, should be considered in future algorithms with volitional bolus swallows because both swallow types are activated in the pre-central motor and post-central somatosensory cortical regions [37], generate pharyngeal and upper oesophageal sphincter pressures [38, 39], and have similar swallow apnoea durations [40]– factors that can influence swallow sound acoustic characteristics of amplitude (loudness) and duration (length). Additionally, spontaneous saliva swallows occur at a rate of one per minute at rest, with increased rates post fluid intake due to increased saliva production [41]. Future swallow sound detection and segmentation algorithms should consider recording of swallow sounds simultaneously with VFSS and fibreoptic endoscopic examination of swallowing (FEES) to objectively classify the presence of both volitional bolus and automated saliva swallows in children.

The successful automation of the detection and identification of the start and end points of swallowing sounds in pediatric feeding disorders opens a new era for the advancement of CA as a diagnostic tool for detecting aspiration worldwide. We previously reported on our machine learned aspiration classifier with 100% precision in children [11]. Combining our aspiration classifier with our automatic detection and segmentation algorithm for swallowing sounds provides important foundations for the development of cervical auscultation into an objective technological application. The development of an application that does not need expert clinicians to provide time-consuming manual segmentation of digitally recorded sounds for cervical auscultation use in the clinical setting potentially increases access to the accurate detection of aspiration– particularly in low-income countries and helps to complement instrumental assessments such as FEES and VFSS. The inclusion of bolus swallow sounds from a range of thickened fluids and teat flow rates are recommended in future algorithm refinements to facilitate meaningful translation of a developed cervical auscultation in clinical practice.

### Further Work and Limitations of the Present Study

While the model we have presented was successful in automatically detecting the location of swallows, there are some limitations and future improvements that are worth mentioning. While we have improved the temporal resolution of the output segments by dividing the large frame length of 0.96 s into subframes, further investigation would be ideal to determine if a larger number of subframes would be optimal. Secondly, the results have shown non-processed, raw segments that have been output from our model, where in some cases there have been false positives and false negatives. It would be worth investigating a suitable postprocessing stage to heuristically decide if the segment output was a swallow or not. This could involve incorporating a priori information such as typical swallow durations, refractory periods, etc. or examining confidence values from the model to determine a suitable hard or soft decision threshold. Thirdly, swallowing problems within paediatrics is heterogeneous and can be transient, developmental, chronic and/or progressive in nature [42]. The automatic segmentation of swallowing sounds has the potential to standardize the detection of swallowing sounds in children; however, future studies on the validation of our algorithm in specific populations is needed. To advance cervical auscultation as a complementary assessment to the VFSS and FEES, the algorithm described in this study could be combined with our previously described aspiration classifier [11] for the development of a digital app for use in clinical practice.

## Conclusion

Our study provides high accuracy (91%) on the automatic segmentation of swallowing sounds in children using transfer machine learning approach from swallowing sounds collected from digital cervical auscultation in children.

## Electronic Supplementary Material

Below is the link to the electronic supplementary material.

Supplementary Material 1

Supplementary Material 2

Supplementary Material 3

Supplementary Material 4

## Data Availability

Ethical approval was not received for sharing of the data for this study.
