# Supplementary material for: Use of Transfer Learning for the Automated Segmentation and Detection of Swallows via Digital Cervical Auscultation in Children
Source: Dysphagia. 2025 Jun 3;40(6):1371–80. doi: 10.1007/s00455-025-10833-3 (PMC12662899; doi:10.1007/s00455-025-10833-3)
Supplement: Supplementary file 1 — Supplementary Material 1 [file 455_2025_10833_MOESM1_ESM.docx]

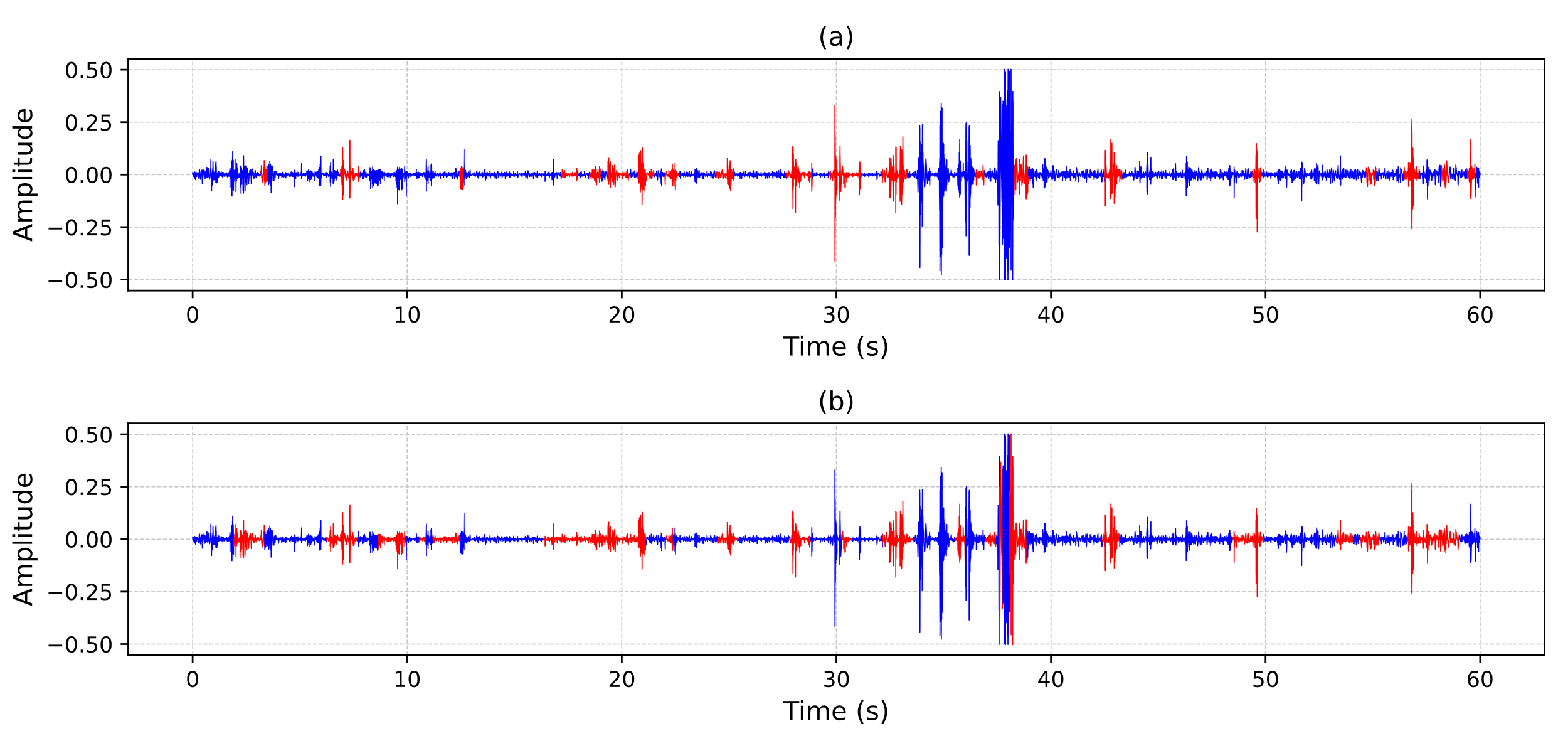


Supplemental Figure 1: Waveforms showing the locations of (a) true labelled swallows (labelled as red); and (b) model-predicted swallows (labelled as red) from VFSS of participant 18 **bottle feeding** on thin fluids in the validation dataset.
