# Supplementary material for: Use of Transfer Learning for the Automated Segmentation and Detection of Swallows via Digital Cervical Auscultation in Children
Source: Dysphagia. 2025 Jun 3;40(6):1371–80. doi: 10.1007/s00455-025-10833-3 (PMC12662899; doi:10.1007/s00455-025-10833-3)
Supplement: Supplementary file 2 — Supplementary Material 2 [file 455_2025_10833_MOESM2_ESM.docx]

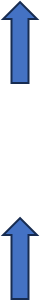

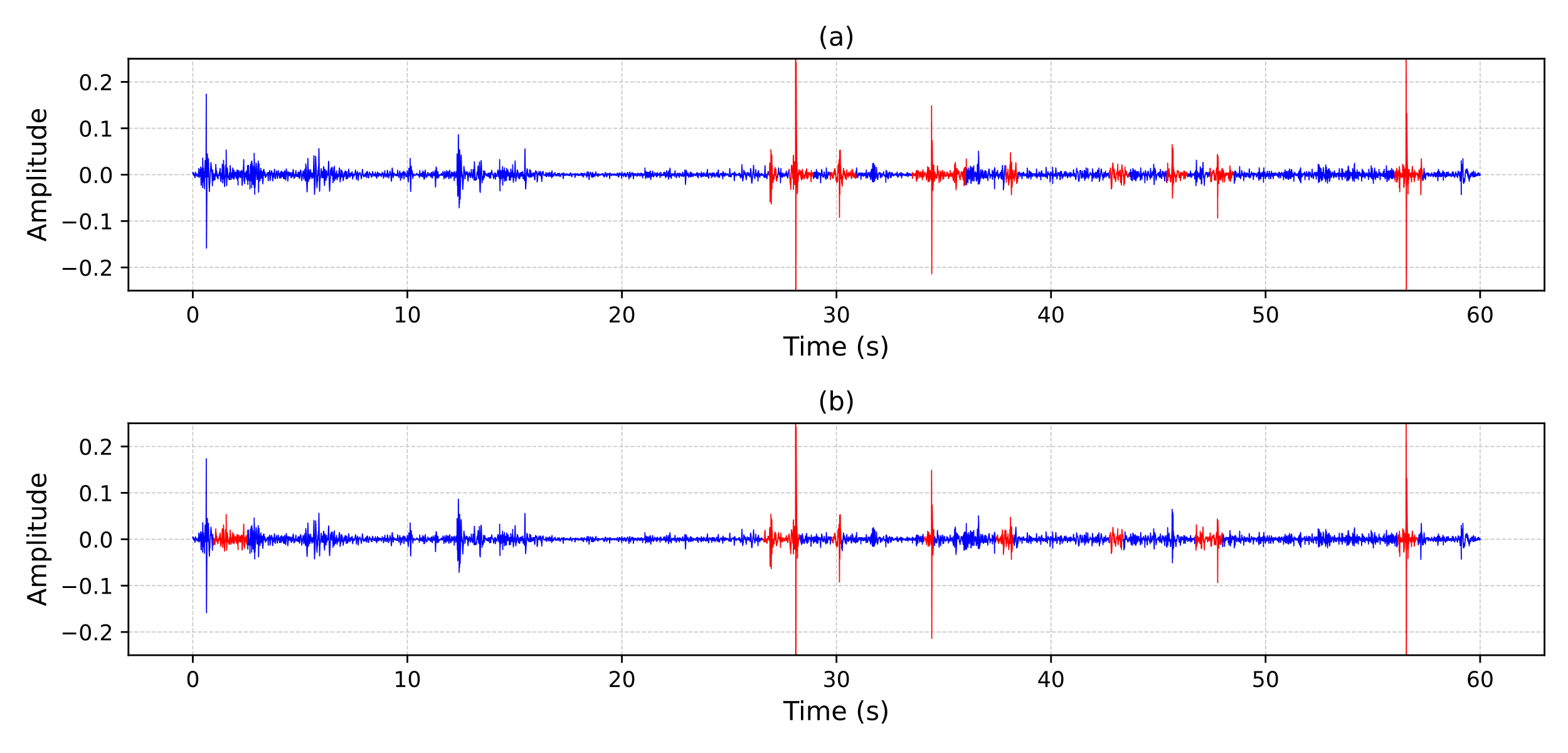


Supplemental Figure 2: Waveforms showing the locations of (a) true swallows (labelled as red); and (b) model-predicted swallows (labelled as red) of participant 14 **cup feeding** on thin fluids in the testing dataset. Saliva swallow is indicated by the blue arrow.
