# Supplementary material for: Use of Transfer Learning for the Automated Segmentation and Detection of Swallows via Digital Cervical Auscultation in Children
Source: Dysphagia. 2025 Jun 3;40(6):1371–80. doi: 10.1007/s00455-025-10833-3 (PMC12662899; doi:10.1007/s00455-025-10833-3)
Supplement: Supplementary file 3 — Supplementary Material 3 [file 455_2025_10833_MOESM3_ESM.docx]

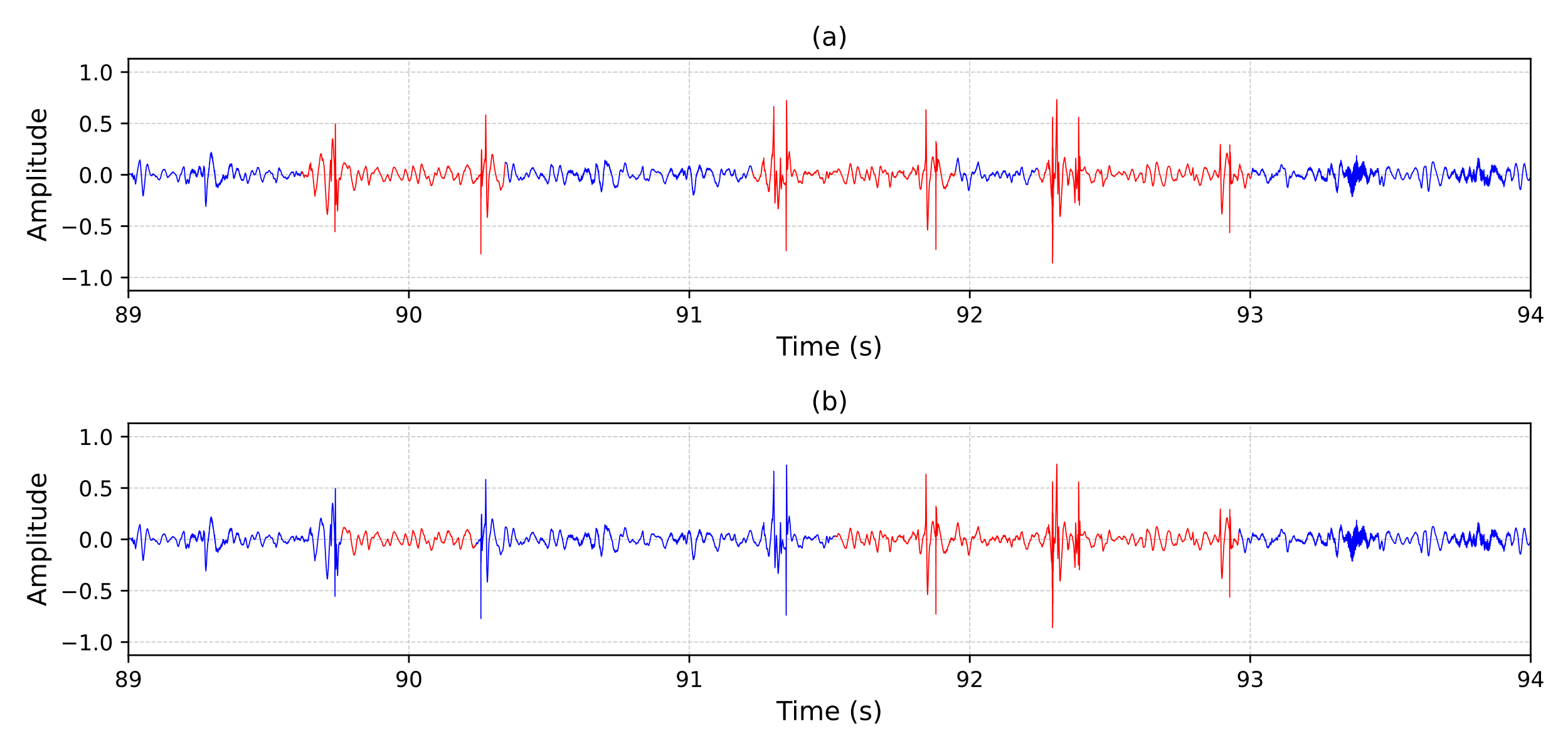


L

C

R

Swallow 1

Swallow 2

Swallow 3

Supplemental Figure 3: A zoomed-in five-second section showing the locations of three (a) true swallows (labelled as red); and (b) model-predicted swallows (labelled as red) of participant 21 in the testing dataset. Swallow 1 has been segmented into left (L), centre (C), and right (R) events.
