# Supplementary material for: Use of Transfer Learning for the Automated Segmentation and Detection of Swallows via Digital Cervical Auscultation in Children
Source: Dysphagia. 2025 Jun 3;40(6):1371–80. doi: 10.1007/s00455-025-10833-3 (PMC12662899; doi:10.1007/s00455-025-10833-3)
Supplement: Supplementary file 4 — Supplementary Material 4 [file 455_2025_10833_MOESM4_ESM.docx]

Supplemental Table 1. Glossary for digital signal processing terms

| Terminology | Definition |
| --- | --- |
| Deep neural network (DNN) | A neural network architecture that contains three or more hidden layers. |
| Fully-connected neural network (FCNN) | Also known as a multiplayer perceptron (MLP), this is a supervised learning model for classification and regression that is biologically-inspired by the interconnection of neurons in the brain. |
| Gaussian mixture model (GMM) | A probabilistic maximum-likelihood parametric framework using a weighted mixture of Gaussian sources to model data. |
| Hidden markov model (HMM) | A probabilistic framework that models data as observations of a unobservable states of some Markov process. |
| Neural network (NN) | A generalised supervised learning model that is biologically-inspired by the interconnection of neurons in the brain. There are many different architectures, such as fully-connected neural networks, convolution neural networks, recurrent neural networks, etc. |
| log-Mel spectrogram | Time-frequency analysis of a signal where frequency has been warped according to the Mel-scale. |
| Mel frequency cepstrum coefficients (MFCC) | Handcrafted features used in previous generation automatic speech recognition systems that attempted to mimic the human auditory system. |
| Mel-scale filter | A filterbank that is spaced according the Mel-scale that is used to mimic the perception of sounds by the human auditory system. |
| Mel-scale warping | A non-linear frequency scale that correlates to perceptually equi-distant pitch. |
| Rectified linear unit (ReLU) | A non-linear activation function used in hidden layer units of a neural network. |
| Support vector machine (SVM) | A supervised learning model for classification that finds the optimum decision surface by maximizing the margin, i.e. minimum distance between support vectors and the decision surface. |
| Zero-crossing rate (ZCR) | A feature used in previous generation automatic speech recognition system that measures the rate of sign changes in a signal. |
